# Combination of Ranibizumab with macular laser for macular edema secondary to branch retinal vein occlusion: one-year results from a randomized controlled double-blind trial

**DOI:** 10.1186/s12886-020-01498-7

**Published:** 2020-06-19

**Authors:** Shuang Song, Xiaobing Yu, Peng Zhang, Xiaoya Gu, Hong Dai

**Affiliations:** Department of Ophthalmology, Beijing Hospital, National Center of Gerontology, Institute of Geriatric Medicine, Chinese Academy of Medical Sciences, Beijing, People’s Republic of China

**Keywords:** Branch retinal vein occlusion, Macular edema, Ranibizumab, Macular grid photocoagulation

## Abstract

**Background:**

It is not clear whether macular laser combined with anti-vascular endothelial growth factor (VEGF) can reduce the number of anti-VEGF injections in the treatment of macular edema (ME) secondary to branch retinal vein occlusion (BRVO). Our study aimed to investigate the effects of intravitreal ranibizumab with or without macular laser for ME secondary to BRVO and its associated number of anti-VEGF injections.

**Methods:**

This is a prospective, randomized, double-blind, monocentric trial.80 patients were enrolled and 64 patients fulfilled the study requirements. All patients received a minimum of 3 initial monthly ranibizumab injections, pro re nata (PRN) dosing thereafter VA and CRT stabilization criteria-driven PRN treatment. Laser was given 7 days after third ranibizumab injection in ranibizumab with laser group. The follow-up time of this study was 1 year. Best corrected visual acuity (BCVA) improvement, central retinal thickness (CRT) reduction and number of injections of patients were compared between two groups. T-test, non-parametric Wilcoxon test and chis-square tests were adopted for between-group comparisons.

**Results:**

Thirty patients received intravitreal ranibizumab 0.5 mg alone and 34 patients received intravitreal ranibizumab 0.5 mg with macular laser. At 52 week, BCVA increased significantly and CRT decreased significantly in both groups (*P* < 0.001). However, there was no significant difference in BCVA improvement with baseline BCVA adjusted (*p* = 0.5226), and in the CRT reduction (*P* = 0.4552) between two groups after 52 weeks. There was also no significant difference in the number of injections between the two groups. (*P* = 0.0756). There was also no significant difference between ischemic and non-ischemic groups in BCVA improvement, CRT reduction and number of injections (*P* > 0.05).

**Conclusions:**

Our study suggests that ranibizumab combined with macular laser is effective in the treatment of ME secondary to BRVO after 1 year of treatment with 3 + PRN regimen. However, combination of macular grid photocoagulation showed no beneficial anatomical or functional effect during follow-up period, nor did it reduce the number of ranibizumab injections, either in ischemic group or non-ischemic group. We suggest that there is no need to combine macular grid photocoagulation in the treatment of ME secondary to BRVO in the future.

**Trial registration:**

Clinical Trials NCT03054766. https://register.clinicaltrials.gov.Prospectively registered.

## Background

Branch retinal vein occlusion (BRVO) is a common sight-threatening retinal vascular disease. The prevalence rate of BRVO is 4.42 cases per 1000 people [1, 2]. Macular edema (ME) secondary to BRVO is considered to be the main cause of visual impairment [3]. Recently, the treatment options for BRVO include anti-vascular endothelial growth factor (VEGF), corticosteroid and macular laser [4, 5].

It is now recognized that anti-vascular endothelial growth factor drugs are first-line treatment, but repeated injections are needed [6], which increases the financial burden of patients. And at present, there is no consensus on the treatment regimen. The clinical trial of globular phase III (BRAVO study) confirmed the efficacy of ranibizumab in the treatment of BRVO. It is recommended that once a month, at least 6 times continuously, follow (6 + PRN) as needed [7]. In 2015, European ophthalmologist published a consensus that monthly injections achieved the best vision, followed by three consecutive follow-up visits (3 + 3PRN) until visual acuity was stabilized [4]. Compared with 6 + PRN regimen, the 3 + 3PRN treatment can reduce the economic burden.

Macular laser for ME secondary to BRVO has been the standard therapy since the 1980s and the BVOS studies indicated that eyes which received macular laser therapy were more likely to maintain reasonable visual acuity when compared to the untreated eyes [8]. The long-term results showed that the beneficial effect of macular laser is obvious, but the treatment response of some patients is not sufficient. Furthermore, the side-effect of macular laser was iatrogenic paracentral scotomas probably [9]. Anti-VEGF drugs did not have the above side effects. Therefore, in the era of anti-VEGF, whether it is necessary for macular laser is worth discussing.

Since both macular laser and ranibizumab can effectively treat macular edema, we inferred that the combination of macular laser and ranibizumab can reduce the number of injections and reduce the economic burden of patients. The purpose of this study was to evaluate whether macular laser combined with ranibizumab injection is more beneficial to ME due to BRVO in terms of functional and anatomical results and reinjection frequency compared with ranibizumab alone. Here, we report the 12-month outcomes of our study (Clinical trials.gov identifier: NCT 03054766).

## Methods

### Trial design

This study was a prospective, double-blind, single-center, randomized clinical trial (1:1 for two groups) which followed the principles of the Declaration of Helsinki and was approved by the Ethics Committee of Beijing Hospital. The study was conducted between February 2017 and August 2019.All participants signed a standard informed consent form reporting on the potential risks, benefits of the procedure, subsequent management and they could not be identified through this document.

### Participants

This study included 64 patients (one eye per patient) finally from enrolled 80 patients who were diagnosed with ME due to BRVO. All patients were confirmed by the ophthalmology department of Beijing Hospital for a comprehensive examination including blood pressure, best corrected visual acuity (BCVA), intraocular pressure (IOP), slit-lamp biomicroscopy, auto refractometry, gonioscopy, optical coherence tomography (OCT), fluorescein angiography (FA) and dilated fundoscopic examinations of both eyes.

The study enrolled treatment-naïve patients older than 18 years of age suffering from ME secondary to BRVO within 12 months, the BCVA letters score at baseline between 24 and 73 Early Treatment Diabetic Retinopathy Study (ETDRS) letters (approximate Snellen chart equivalent of 20/400 and 20/40) and the Central retinal thickness (CRT) was more than 250um.The patients were then classifed into two groups based on the presence or absence of retinal non-perfusion on FFA [10]. Patients were excluded if they met the following criteria: (1) hemi-CRVO or CRVO (2) diabetic maculopathy and/or retinopathy; (3) any other BCVA compromising ocular disease; (4) any prior intravitreal anti-VEGF or corticosteroid injections; (5) any prior retinal laser photocoagulation; (6) IOP higher than 21 mmHg; (7) history of vitrectomy; (8) history of myocardial infarction or stroke with 3 months; and (9) other major systemic disorders.

### Randomization and interventions

Shuang Song generated the random allocation sequence and Peng Zhang enrolled participants and assigned participants to interventions.

All eligible patients were randomly assigned (1:1) according to random number table to receive intravitreal ranibizumab 0.5 mg (ranibizumab monotherapy group) or intravitreal ranibizumab 0.5 mg with laser (IVR + Laser group). BCVA and CRT were the primary trigger of retreatment. Patients and investigators responsible for the treatment were both masked and the decision of re-treatment was based on the changes of BCVA and CRT. The eyes with more than 5 letters (ETDRS) loss due to disease activity or more than 100um increase of CRT would receive re-treatment every 4 weeks. The treatment protocol was similar to Gu’s study [11].

The patients of IVR + laser group would receive macular grid laser photocoagulation 7 days after the third injection. Laser application was performed by Doctor Yu with a pan-funduscopic TransEquator lens (Volk optical Inc., Mentor, Ohio, USA) (spot size 100 μm; energy 100-300 mW; exposure time 100 msec; Volk Goldmann lense®) until soft whitening of the retina became apparent, according to the physician’s discretion. The patients of IVR group would receive sham macular grid laser photocoagulation 7 days after the third injection. Therefore, all patients who attained the criteria of re-treatment would receive re-injection of ranibizumab during follow-up period.

### Study objectives

Our primary objective was to evaluate the change in BCVA and CRT in both groups after 52 weeks. Secondary objectives were to analyze the number of injections up to week 52 and the interval time from third to fourth injection.

### Outcomes

Best corrected visual acuity (BCVA) improvement, central retinal thickness (CRT) reduction and the number of ranibizumab injections.

### Best-corrected vision acuity

BCVA of patients was assessed with ETDRS VA testing charts by a certified examiner at baseline and every follow-up visit. The standard testing distance was 4 m, changing to 1 meter in case a patient could not read 4 letters at 4 m at least [12].

### Optical coherence tomography

SD-OCT (Heidelberg, Germany) examinations were conducted at every visit. Central retinal thickness (CRT) was measured by automated measurements provided by OCT software to measure central retinal thickness automatically.

### Efficacy and safety assessments

The incidence of no matter ocular or non-ocular adverse events (AEs) and severe adverse events (SAEs) was assessed during follow-up period, and to indicate their possible relationship to ocular intravitreal injections and/or the study therapy.

### Statistical analysis

Statistical analyses were performed with SAS 9.4 (SAS Institute Inc. Cary, North Carolina, USA) and statistical significance was established at two-tailed *p* < 0.05. Data were summarized as number (percentage), Mean ± Standard Deviation (SD) or Median (interquartile ranges [IQR]) as appropriate. T-test or non-parametric Wilcoxon test were adopted for between-group comparisons in normally or non-normally distributed continuous variables and chis-square tests for categorical variables. To analyze changes in BCVA as well as CRT, general linear models were constructed for variance analysis with baseline BCVA adjusted. Kaplan-Meier and cox proportional hazard models with covariates were constructed to compare time to the fourth injection after three initial injections. Safety analyses were conducted on the safety set. Adverse events (AEs) were summarized by reporting the number and percentage of patients with any ocular and/or non-ocular AEs.

## Results

### Baseline demographics and ocular characteristics of participants

Eighty patients were screened and 64 patients completed the trial finally.30 (46.9%) patients were randomized into IVR group and 34 (53.1%) patients were enrolled into IVR + Laser group. According to the condition of ischemia, the above two groups were divided into the following two subgroups: In IVR group, there were 11 eyes in ischemic group and 19 eyes in non-ischemic group. In IVR + Laser group, there were 16 eyes in ischemic group and 18 eyes in non-ischemic group. Each group’s baseline characteristics are displayed in Table 1. Baseline BCVA was unbalanced between two groups with *p* value of less than 0.05.

**Table 1:** Baseline demographics and ocular characteristics of participants

Variables	IVR (*N* = 30)	IVR + Laser (*N* = 34)	*P* value
Age, years	59.6 ± 11.0	58.4 ± 9.7	0.6392
Gender, male	17 (56.7%)	18 (52.9%)	0.7651
Ischemic	11 (36.7%)	16 (47.1%)	0.4009
BCVA	59.3 ± 8.3	54.4 ± 9.8	0.0384^*^
CRT (μm)	516.1 ± 161.1	571.6 ± 223.5	0.2647
SBP (mmHg)	136.9 ± 11.6	131.5 (127, 140)	0.3457
DBP (mmHg)	79.3 ± 8.7	79.4 ± 10.8	0.9548
Intraocular pressure (mmHg)	16.2 ± 2.3	15.5 ± 2.6	0.2919

*BCVA* best corrected visual acuity, *CRT* central retinal thickness, *SBP* systolic blood pressure, *DBP* diastolic blood pressure

^*^P < 0.05

### Visual outcomes and anatomical outcomes

**Fig. 1:**
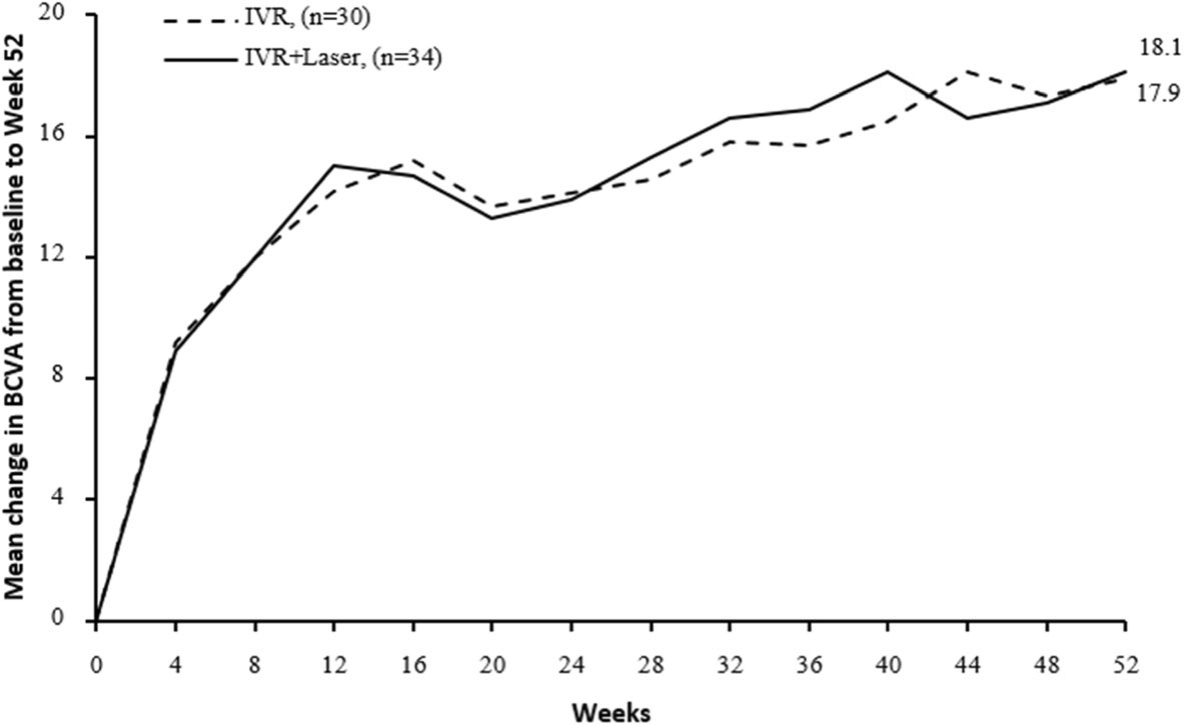
Mean change in BCVA from baseline to the last visit. In comparison between two groups, general linear model was constructed as well for variance analysis with baseline BCVA adjusted. Mean gains in BCVA from baseline to week 52 were similar between two groups (*P* = 0.5226). Change in BCVA from baseline to week 52 were normally distributed (*P* > 0.05 in Shapiro-Wilk tests in both groups). Mean ± SD change in BCVA from baseline to week 52 was 17.9 ± 9.0 letters in IVR group, 18.1 ± 9.9 letters in IVR + Laser group

**Fig. 2:**
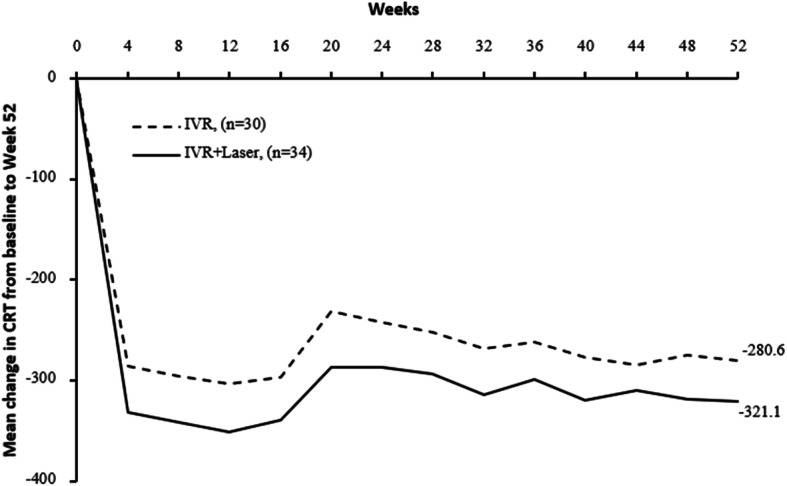
Mean change in CRT from baseline to the last visit. In comparison between two groups, general linear model was constructed as well for variance analysis with baseline CRT adjusted. Mean gains in CRT from baseline to week 52 were similar between two groups (*P* = 0.3999). Change in CRT from baseline to week 52 were normally distributed (P > 0.05 in Shapiro-Wilk tests in both groups). Mean ± SD change in CRT from baseline to week 52 was − 280.6 ± 181.4 μm in IVR group, − 321.1 ± 240.5 μm in IVR + Laser group

The changes of BCVA and CRT between two groups (IVR vs. IVR + Laser) were showed in Figs. 1 and 2 and Appendix 1 and 2. Significant differences in BCVA (ETDRS) at baseline between two groups were observed (*P* < 0.05). At 52 week, we noted a significant gain in BCVA in both groups (*P* < 0.001) (Appendix 3). However, there was no significant difference in BCVA improvement (17.9 letters vs. 18.1 letters) between two groups with baseline BCVA adjusted after 52 weeks (*p* = 0.5226). The BCVA improved steeply during the first 3 month but gradually from week 12 to week 52 as shown in Fig. 1. Comparing two groups, BCVA improved both 14 letters at week 24 but increased differently 17.9 letters vs. 18.1 letters at week 52 (Appendix 3). No significant differences in CRT measurements were evident at baseline between two groups (*P* = 0.2647). Both groups suggested a significant decrease in CRT after 52 weeks (*P* < 0.001) (Appendix 3). The CRT also decreased steeply during the first 3 month but gradually from week 12 to week 52 as shown in Fig. 2. Comparing two groups, CRT decreased much in IVR+ laser group. However, there was no significant difference in the CRT reduction (280.6 v.s.321.1 μm) between two groups after 52 weeks (*P* = 0.4552) (Appendix 3).

**Fig. 3:**
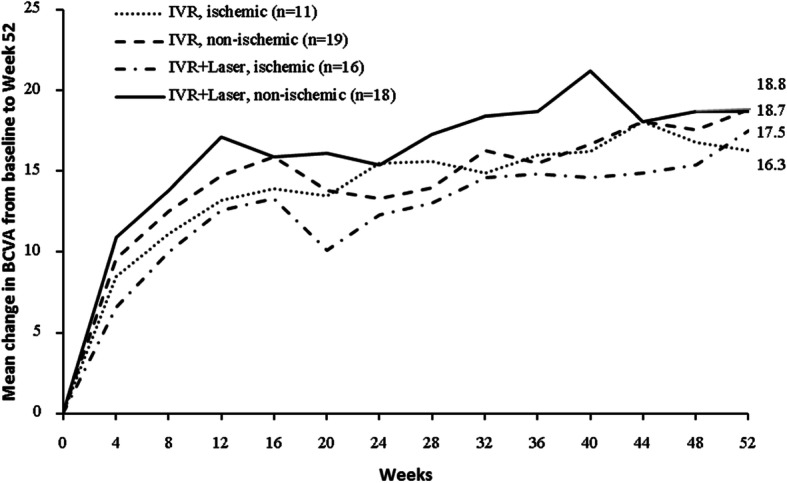
Mean change in BCVA from baseline to the last visit by baseline ischemia. BCVA, best corrected visual acuity; IVR, intravitreal ranibizumab; General linear models were constructed for variance analysis with baseline BCVA adjusted, mean gains in BCVA from baseline to week 52 were similar between two groups in patients with ischemia (*P* = 0.9830) or without ischemia at baseline (*P* = 0.5050). Change in BCVA from baseline to week 52 in patients with or without ischemia were normally distributed (all *P* > 0.05 in Shapiro-Wilk tests). Mean ± SD change in BCVA from baseline to week 52 in patients with ischemia was 16.3 ± 10.3 letters in IVR group, 17.5 ± 8.8 letters in IVR + Laser group. Mean ± SD change in BCVA at week 52 from baseline in patients without ischemia was 18.8 ± 8.2 letters in IVR group, 18.7 ± 11.0 letters in IVR + Laser group

**Fig. 4:**
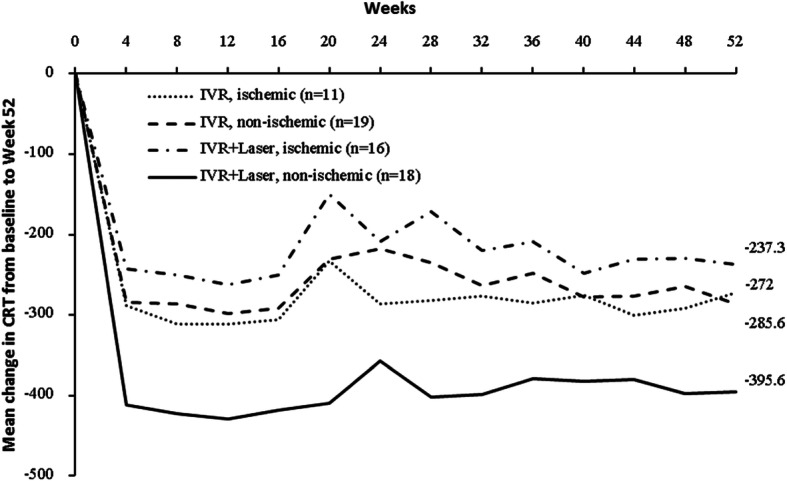
Mean change in CRT from baseline to the last visit by baseline ischemia. CRT, central retinal thickness; IVR, intravitreal ranibizumab; In general, linear model with treatments in two groups and baseline CRT included, mean gains in CRT from baseline to week 52 were not statistically different between two groups in patients with ischemia (*P* = 0.7749) or without ischemia at baseline (*P* = 0.3901). Change in CRT from baseline to week 52 in patients with or without ischemia were normally distributed (all P > 0.05 in Shapiro-Wilk tests). Mean ± SD change in CRT from baseline to week 52 in patients with ischemia was − 272.0 ± 240.1 μm in IVR group, − 237.3 ± 212.3 μm in IVR + Laser group. Mean ± SD change in CRT at week 52 from baseline in patients without ischemia was − 285.6 ± 144.6 μm in IVR group and − 395.6 ± 245.0 μm in IVR + Laser group

The changes of BCVA and CRT between four groups based on ischemic or non-ischemic were showed in Figs. 3 and 4 and Appendix 4 and 5. Similarly, there was no significant difference in BCVA improvement between four groups (ischemic BRVO received ranibizumab alone, non-ischemic BRVO received ranibizumab alone, ischemic BRVO received ranibizumab + Laser and non-ischemic BRVO received ranibizumab+Laser) no matter whether obtained ischemic or not with baseline BCVA adjusted after 52 weeks (*P* > 0.05) (Appendix 6 and 7). Also, there was no significant difference in the CRT reduction between ischemic and non-ischemic groups after 52 weeks (P > 0.05) (Appendix 6 and 7).

### Ranibizumab injections

The median number of injections was 3 (rang 3–4) vs. 4 (range 3–6) in IVR group at 24 weeks vs. 52 week and the number was 4 (range 3–4) vs. 6 (range 3–7) in IVR+ Laser group. Table 2 illustrated the number of injections in both groups at 24 week and 52 week. Though there was no significant difference between groups in the number of injections (*P* = 0.0756), the eyes of IVR + Laser group received more injections no matter at 24 week or 52 week. Though, the time to fourth injection after the third injection did not differ significantly between groups (*P* = 0.1193), the interval was much longer for IVR group (Table 3). By the Kaplan-Meier and cox proportional hazard models with covariates, both basic BCVA and laser were not the risk factor to internal extension between third and fourth injection (Table 4 and Appendix 8–10).

**Table 2:** Number of injections within 6 months and 12 months between two groups

Variable Median (IQR)^a^	IVR (*N* = 30)	IVR + Laser (*N* = 34)	*P* value for group difference
Number of injections within 6 months	3 (3, 4)	4 (3, 4)	0.1046
Number of injections within 12 months	4 (3, 6)	6 (3, 7)	0.0756

^a^As injections during follow up were abnormally distributed, median (IQR) and Wilcoxon analysis were used

**Table 3:** Time to fourth injection between two groups

Variable Median (IQR)^a^	IVR (*N* = 30)	IVR + Laser (*N* = 34)	*P* value for group difference
Time to fourth injection	28 (8, 40)	12 (8, 40)	0.1056

^a^As time to fourth injection in two groups were abnormally distributed, median (IQR) and Wilcoxon analysis were used

**Table 4:** Analysis for time to fourth injection during follow-up in Cox regression

Variable	HR	95%CI	*P* value
Group (ref = 1)			
Group = 2	1.43	(0.75, 2.76)	0.2797
BCVA at baseline	0.98	(0.95, 1.02)	0.3330

*BCVA* best corrected visual acuity

Table 5 illustrated the number of injections in four groups based on ischemic or not at 24 week and 52 week. The non-ischemic groups received more injections than ischemic groups both at 24 weeks and 52 weeks though there were no significant difference between ischemic and non-ischemic groups after 52 weeks (all *P* > 0.05). The eyes of IVR + Laser group received more injections at 24 week and 52 week no matter with ischemic or not.

**Table 5:** Number of injections within 6 months and 12 months between ischemia and non-ischemia in each group

Variable		Non-ischemic (*N* = 37)		Ischemic (*N* = 27)		*P* value for differences in injection numbers between non-ischemia and ischemia^a^
Median (Quartile)		N	Injection numbers	N	Injection numbers	
Within 6 months	IVR	19	4 (3, 4)	11	3 (3, 4)	0.4026
	IVR + Laser	18	4 (4, 4)	16	3.5 (3, 4.5)	0.2967
Within 12 months	IVR	19	4 (3, 6)	11	3 (3, 7)	0.9273
	IVR + Laser	18	6 (6, 7)	16	4 (3, 6.5)	0.1274

^a^As injections during follow up were abnormally distributed, median (IQR) and Wilcoxon analysis were used

### Adverse events

Four patients (3 in IVR group and 1 in IVR+ laser group) exhibited IOP increase during study. IOP return to normal levels with topical therapy. Two patients withdrew the study because of lacunar infarction, then they received appropriate medical treatment to keep health. No other AEs or SAEs of any kind were recorded during study period.

## Discussion

Both ranibizumab monotherapy and ranibizumab with laser therapy could improve BCVA and decrease CRT significantly in ME patients due to BRVO from this study. However, our results indicated that the effect between ranibizumab monotherapy and ranibizumab with laser were similar in no matter functional or anatomical benefit during 1 year. Also, the number of injections was similar (4 vs. 6) between two groups (*P* = 0.0756). Some recent reports have also shown that this combination therapy can significantly improve BCVA and reduce CRT [12–15], but the number of injections has not decreased, which is similar to the results of our study [12, 13]. In the past we thought ranibizumab can neutralize upregulated intravitreal VEGF levels which contribute to ME development due to a blood-barrier breakdown in BRVO [16], and laser can activate the pump function of retinal pigment epithelium and transport fluid out of the retinal structures to reduce CRT due to ME [17]. Therefore, the combination therapy of ranibizumab with Laser in the treatment of macular edema has the above pathophysiological theoretical basis. Based on the above, we hypothesized that the combination of ranibizumab and laser therapy may reduce the number of injections. However, our results show that combination of macular grid photocoagulation showed no beneficial anatomical or functional effect during follow-up period, nor did it reduce the number of ranibizumab injections.

Our results showed that there was no significant difference in the injection interval (the interval between the third and fourth injections) after combined macular grid photocoagulation, and there was no significant difference in the number of injections between the two groups within 1 year. The results showed that combined macular grid photocoagulation could not prolong the injection interval in the short term and could not reduce the injection times in the long term. We speculate that it may be related to the increase of intraocular inflammatory factors in the short term after laser treatment. So it needs to be injected more times to inhibit these inflammatory factors, since ranibizumab can not only inhibit VEGF but also inhibit inflammatory factors [18, 19]. There is only one macular laser in this study, it is speculated that the more times of macular laser, the more times of injection may be needed, so combined macular grid photocoagulation is not recommended.

Based on the above results, considering the retinal damage caused by macular laser and the economic burden of patients, we suggest that there is no need to combine macular grid photocoagulation in the treatment of macular edema secondary to BRVO in the future. From two-years results of the BRIGHTER study, ranibizumab was initially applied three times a monthly, followed by a VA stabilization criteria-driven PRN treatment regimen. Laser was performed on the same day ≥30 min before ranibizumab injection in the combination group; they observed a significant BCVA gain and CRT reduction but there was no significant difference between the two groups, and there was no difference in the number of injections [13]. In their retrospective analysis, Farese showed that the combination therapy was more effective and required fewer injections. Laser was applied 2 weeks after bevacizumab injection [20]. They indicated that the strongest bevacizumab effect and CRT reduction was 2 weeks after anti-VEGF injections and that might be the most effective time point for laser treatment [20]. Their results showed that the average number of injections was 2.73 in combination therapy and 3.13 in bevacizumab alone therapy [20]. In our study, ranibizumab was also administered three times on a monthly basis, followed by a VA and CRT stabilization criteria-driven PRN treatment regimen, and Laser was performed 1 week after the third injection. We thought that the decrease of CRT is the most obvious after three injections of ranibizumab, and it is the best to be treated with laser 1 week after the third injection. As shown in Appendix 6 and 7 of this study, although there was a significant difference in the reduction of macular edema between the two groups within 1 year (285.6 vs 395.6 μm), there was no significant change in visual acuity (18.8 vs.18.7). Therefore, we believe that the improvement of visual acuity is not completely proportional to the reduction of macular edema, indicating that both BCVA and CRT may be more appropriate as criteria for retreatment. In addition, the efficacy time of bevacizumab was 6 weeks, while that of ranibizumab was 4 weeks [20]. These differences may lead to more injections in our studies. Different anti-VEGF efficacy time and different retreatment criteria may be the reasons for the different results of our study and Farese study [20].

Our results showed that there was no difference in improvement of visual acuity, reduction of macular edema and injection times within 1 year regardless of whether ischemia or whether combined with laser therapy. In previous similar studies, Callizo focused on BRVO patients with non-ischemic macular edema [12], while Tadayoni divided BRVO patients into macular ischemic and non-ischemic groups [13]. These above are different from our grouping criteria, in which BRVO patients are grouped according to whether the retina is ischemic or not, and macular ischemia is not involved. In the past, it was considered that the visual acuity prognosis of retinal non-ischemic BRVO was better than that of ischemic type, and that of macular non-ischemic BRVO was better than that of ischemic type [13, 21]. However, recent studies have shown that with the intervention of anti-VEGF therapy, the visual prognosis of ischemic and non-ischemic BRVO is the same [13]. It is speculated that the possible reason is that the level of VEGF in macular edema due to BRVO is significantly increased. After 3 consecutive anti-VEGF treatments, the level of VEGF in BRVO of ischemic group and non-ischemic group decreased significantly, macular edema could disappear quickly, and visual acuity could be recovered quickly, which may be the reason for the same visual acuity prognosis in both groups. In addition, after three consecutive anti-VEGF treatments combined with macular grid photocoagulation, the laser may further reduce VEGF, but the effect is weak, which may be the reason for the same number of injections between the two groups.

The main limitation of this study is the number of patients is relatively small and the single center. Second,the follow-up period is only 1 year, which is relatively short. Therefore, larger number of patients is needed to be followed up for a longer time to confirm this result in the future.

## Conclusion

Our study suggests that ranibizumab combined with macular laser is effective in the treatment of macular edema secondary to BRVO after 1 year of treatment with 3 + PRN regimen. However, combination of macular grid photocoagulation showed no beneficial anatomical or functional effect during follow-up period, nor did it reduce the number of ranibizumab injections, either in ischemic group or non-ischemic group. We suggest that there is no need to combine macular grid photocoagulation in the treatment of macular edema secondary to BRVO in the future.

## Supplementary information

**Additional file 1: Appendix 1.** Changes of BCVA from baseline to week 52 (Mean ± SD). **Appendix 2.** Changes of CRT from baseline to week 52 (Mean ± SD). **Appendix 3.** BCVA and CRT differences from baseline to the last visit. **Appendix 4.** Absolute value of BCVA from baseline to week 52 by baseline ischemia (Mean ± SD). **Appendix 5.** Absolute value of CRT from baseline to week 52 by baseline ischemia (Mean ± SD). **Appendix 6.** BCVA and CRT differences from baseline to the last visit by ischemia. **Appendix 7.** Changes of BCVA and CRT from baseline to the last visit between non-ischemia and ischemia by group. **Appendix 8.** Product-Limit Survival Estimates in group 1. **Appendix 9.** Product-Limit Survival Estimates in group 2. **Appendix 10.** Time to fourth injection after the third injection between two groups.

## Data Availability

The datasets used and/or analyzed during the current study are available from the corresponding author upon reasonable request.

## Abbreviations

BCVA: Best corrected visual acuity
BRVO: Branch retinal vein occlusion
BVOS: Branch Vein Occlusion Study
CRT: Central retinal thickness
DBP: Diastolic blood pressure
ETDRS: Early Treatment Diabetic Retinopathy Study
Laser: Macular grid laser photocoagulation
ME: Macular edema
OCT: Optical coherence tomography
PRN: Pro re nata
SBP: Systolic blood pressure
VA: Visual acuity
VEGF: Vascular endothelial growth factor
