# Supplementary material for: Combination of Ranibizumab with macular laser for macular edema secondary to branch retinal vein occlusion: one-year results from a randomized controlled double-blind trial
Source: BMC Ophthalmol. 2020 Jun 19;20:241. doi: 10.1186/s12886-020-01498-7 (PMC7304204; doi:10.1186/s12886-020-01498-7)
Supplement: Supplementary file 1 — Additional file 1: Appendix 1. Changes of BCVA from baseline to week 52 (Mean ± SD). Appendix 2. Changes of CRT from baseline to week 52 (Mean ± SD). Appendix 3. BCVA and CRT differences from baseline to the last visit. Appendix 4. Absolute value of BCVA from baseline to week 52 by baseline ischemia (Mean ± SD). Appendix 5. Absolute value of CRT from baseline to week 52 by baseline ischemia (Mean ± SD). Appendix 6. BCVA and CRT differences from baseline to the last visit by ischemia. Appendix 7. Changes of BCVA and CRT from baseline to the last visit between non-ischemia and ischemia by group. Appendix 8. Product-Limit Survival Estimates in group 1. Appendix 9. Product-Limit Survival Estimates in group 2. Appendix 10. Time to fourth injection after the third injection between two groups. [file 12886_2020_1498_MOESM1_ESM.docx]

**Appendix 1. Changes of BCVA from baseline to week 52 (Mean±SD)**

| **BCVA** | **Baseline** | **Week 4** |  | **Week 8** | **Week 12** | **Week 16** | **Week 20** | **Week 24** | **Week 28** | **Week 32** | **Week 36** | **Week 40** | **Week 44** | **Week 48** | **Week 52** |
| --- | --- | --- | --- | --- | --- | --- | --- | --- | --- | --- | --- | --- | --- | --- | --- |
| IVR (n=30) | 59.3±8.3 | 68.5±11.4 |  | 71.2±12.0 | 73.4±11.2 | 74.5±10.8 | 73.0±10.1 | 73.4±11.3 | 73.9±12.6 | 75.1±11.7 | 74.9±11.2 | 75.8±10.7 | 77.3±9.9 | 76.6±11.8 | 77.1±11.0 |
| IVR+Laser (n=34) | 54.4±9.8 | 63.3±10.6 |  | 66.4±10.5 | 69.4±10.2 | 69.1±10.5 | 67.7±11.4 | 68.4±10.2 | 69.7±10.8 | 71.1±11.0 | 71.3±11.1 | 72.5±10.5 | 71.0±12.6 | 71.6±10.3 | 72.6±10.2 |

**Appendix 2. Changes of CRT from baseline to week 52 (Mean±SD)**

| **CRT** | **Baseline** | **Week 4** | **Week 8** | **Week 12** | **Week 16** | **Week 20** | **Week 24** | **Week 28** | **Week 32** | **Week 36** | **Week 40** | **Week 44** | **Week 48** | **Week 52** |
| --- | --- | --- | --- | --- | --- | --- | --- | --- | --- | --- | --- | --- | --- | --- |
| IVR (n=30) | 516.1±161.1 | 230.7±52.9 | 221.0±62.7 | 213.4±38.5 | 219.4±45.6 | 284.9±106.4 | 273.7±98.1 | 264.2±83.4 | 248.3±55.0 | 254.3±71.5 | 239.6±47.0 | 231.2±48.0 | 241.4±59.4 | 235.5±62.9 |
| IVR+Laser (n=34) | 571.6±223.5 | 239.7±114.4 | 229.9±77.9 | 220.8±75.6 | 232.7±82.9 | 284.4±145.3 | 284.5±108.9 | 277.9±137.9 | 257.3±97.4 | 272.5±114.2 | 252.2±84.9 | 262.1±92.4 | 252.9±103.0 | 250.5±85.0 |

**Appendix 3. BCVA and CRT differences from baseline to the last visit**

| Variable  Mean±SD | IVR  (N=30) | | | |  | IVR+Laser  (N=34) | | | | P value for group difference |
| --- | --- | --- | --- | --- | --- | --- | --- | --- | --- | --- |
|  | Baseline | Week 52 | Within group change, mean (95% CI) | P value for change |  | Baseline | Week 52 | Within group change, mean (95% CI) | P value for change |  |
| BCVA | 59.3±8.3 | 77.1±11.0 | 17.9 (14.5, 21.2) | <0.0001 |  | 54.4±9.8 | 72.6±10.2 | 18.1 (14.7, 21.6) | <0.0001 | 0.5226^*^ |
| CRT（μm） | 516.1±161.1 | 235.5±62.9 | -280.6 (-348.3, -212.9) | <0.0001 |  | 571.6±223.5 | 250.5±85.0 | -321.1 (-405.0, -237.1) | <0.0001 | 0.4552 |

BCVA, best corrected visual acuity; CRT, central retinal thickness; ^*^ As baseline BCVA was unbalanced between groups, general linear model with baseline BCVA adjusted was performed for group difference analysis. In univariate analysis without adjusting baseline BCVA, P value for group difference was 0.9063.

**Appendix 4. Absolute value of BCVA from baseline to week 52 by baseline ischemia (Mean±SD)**

| **BCVA** | **Baseline** | **Week 4** | **Week 8** | **Week 12** | **Week 16** | **Week 20** | **Week 24** | **Week 28** | **Week 32** | **Week 36** | **Week 40** | **Week 44** | **Week 48** | **Week 52** |
| --- | --- | --- | --- | --- | --- | --- | --- | --- | --- | --- | --- | --- | --- | --- |
| IVR | | | | | | | | | | | | | | |
| Ischemic (n=11) | 58.9±7.7 | 67.5±10.4 | 70.0±12.4 | 72.1±11.5 | 72.8±11.2 | 72.5±10.9 | 74.5±12.3 | 74.5±12.7 | 73.8±12.8 | 74.9±12.4 | 75.1±11.8 | 77.0±11.2 | 75.7±14.6 | 75.2±13.1 |
| Non-ischemic (n=19) | 59.5±8.8 | 69.1±12.2 | 71.9±12.1 | 74.2±11.3 | 75.4±10.8 | 73.3±9.9 | 72.8±11.0 | 73.5±12.9 | 75.8±11.3 | 74.9±10.8 | 76.2±10.4 | 77.5±9.5 | 77.1±10.2 | 78.3±9.7 |
| IVR+Laser | | | | | | | | | | | | | | |
| Ischemic (n=16) | 53.7±7.9 | 60.3±9.0 | 63.7±7.8 | 66.3±8.8 | 67.0±9.9 | 63.8±10.8 | 65.9±9.2 | 66.7±11.0 | 68.3±10.2 | 68.5±10.7 | 68.3±10.0 | 68.6±12.6 | 69.1±10.3 | 71.2±9.5 |
| Non-ischemic (n=18) | 55.1±11.4 | 66.1±11.3 | 68.9±12.1 | 72.2±10.8 | 71.1±10.8 | 71.2±11.1 | 70.6±10.8 | 72.4±10.2 | 73.6±11.3 | 73.8±11.3 | 76.3±9.7 | 73.2±12.5 | 73.8±10.2 | 73.8±10.9 |

**Appendix 5. Absolute value of CRT from baseline to week 52 by baseline ischemia (Mean±SD)**

| **CRT** | **Baseline** | **Week 4** | **Week 8** | **Week 12** | **Week 16** | **Week 20** | **Week 24** | **Week 28** | **Week 32** | **Week 36** | **Week 40** | **Week 44** | **Week 48** | **Week 52** |
| --- | --- | --- | --- | --- | --- | --- | --- | --- | --- | --- | --- | --- | --- | --- |
| IVR | | | | | | | | | | | | | | |
| Ischemic (n=11) | 517.6±193.7 | 229.6±67.6 | 206.9±43.5 | 206.8±44.4 | 211.7±44.6 | 284.5±113.0 | 231.4±51.2 | 236.3±73.7 | 241.6±51.6 | 232.5±43.7 | 242.3±40.8 | 217.0±43.2 | 225.5±45.3 | 245.6±89.0 |
| Non-ischemic (n=19) | 515.2±144.8 | 231.3±44.4 | 229.2±71.4 | 217.2±35.3 | 223.9±46.7 | 285.1±105.5 | 298.2±111.0 | 280.4±86.2 | 252.2±57.9 | 266.9±81.9 | 238.1±51.2 | 239.4±49.8 | 250.6±65.6 | 229.6±43.2 |
| IVR+Laser | | | | | | | | | | | | | | |
| Ischemic (n=16) | 495.8±189.0 | 253.3±160.8 | 245.8±103.8 | 233.3±102.3 | 246.3±110.3 | 345.7±189.2 | 287.0±132.0 | 323.9±177.5 | 276.0±129.4 | 287.1±139.5 | 247.4±111.2 | 265.4±107.4 | 266.0±135.0 | 258.6±114.4 |
| Non-ischemic (n=18) | 638.9±235.1 | 227.7±47.5 | 215.8±42.9 | 209.7±39.9 | 220.7±47.7 | 229.8±52.0 | 282.3±87.3 | 237.1±73.2 | 240.7±54.9 | 259.6±88.1 | 256.5±55.1 | 259.1±79.9 | 241.3±65.0 | 243.4±48.6 |

**Appendix 6. BCVA and CRT differences from baseline to the last visit by ischemia**

| Variable  Mean ± SD | | IVR  (N=30) | | | | | | IVR+Laser  (N=34) | | | | | | P value for group difference |
| --- | --- | --- | --- | --- | --- | --- | --- | --- | --- | --- | --- | --- | --- | --- |
|  |  | N | Baseline | Week 52 | Within group change | Within group change, mean (95% CI) | P value for change | N | Baseline | Week 52 | Within group change | Within group change, mean (95% CI) | P value for change |  |
| BCVA | non-ischemic | 19 | 59.5±8.8 | 78.3±9.7^*^ | 18.8±8.2 | 18.8 (14.8, 22.8) | <0.0001 | 18 | 55.1±11.4 | 73.8±10.9 | 18.7±11.0 | 18.7 (13.3, 24.2) | <0.0001 | 0.5050^**^ |
|  | ischemic | 11 | 58.9±7.7 | 75.2±13.1 | 16.3±10.3 | 16.3 (9.3, 23.2) | 0.0004 | 16 | 53.7±7.9 | 71.2±9.5 | 17.5±8.8 | 17.5 (12.8, 22.2) | <0.0001 | 0.9830^**^ |
| CRT（μm） | non-ischemic | 19 | 515.2±144.8 | 229.6±43.2^*^ | -285.6±144.6 | -285.6 (-355.3, -215.9) | <0.0001 | 18 | 638.9±235.1 | 243.4±48.6^*^ | -395.6±245.0 | -395.6 (-517.4, -273.7) | <0.0001 | 0.1102 |
|  | ischemic | 11 | 517.6±193.7 | 245.6±89.0^*^ | -272.0±240.1 | -272.0 (-433.3, -110.7) | 0.0037 | 16 | 495.8±189.0 | 258.6±114.4^*^ | -237.3±212.3 | -237.3 (-350.4, -124.1) | 0.0004 | 0.6952 |

BCVA, best corrected visual acuity; CRT, central retinal thickness; ^*^ The values were abnormally distributed in Shapiro-Wilk test for normality. For non-ischemic patients in IVR group , median (IQR) for BCVA at week 52 was 80 (75, 84); CRT at week 52 were also abnormally distributed in non-ischemic and ischemic patients in both groups, for patients in IVR group, the median (IQR) of CRT in non-ischemic and ischemic patients were 222 (201, 251) μm and 224 (192, 252) μm, respectively; for patients in IVR+Laser group, CRT at week 52 in non-ischemic and ischemic patients were 231.5 (218, 252) μm, and 223.5 (190.5, 315) μm, respectively.^**^ As baseline BCVA was unbalanced between groups, general linear model with baseline BCVA adjusted was performed for group difference analysis. In univariate analysis without adjusting baseline BCVA, P value for group difference was 0.9833 in non-ischemic patients and 0.7425 in ischemic patients.

**Appendix 7. Changes of BCVA and CRT from baseline to the last visit between non-ischemia and ischemia by group**

| Variable  Mean ± SD | | Non-ischemic  （N=37） | | | | | | Ischemic  （N=27） | | | | | P value for differences in changes between non-ischemia and ischemia^*^ |
| --- | --- | --- | --- | --- | --- | --- | --- | --- | --- | --- | --- | --- | --- |
|  |  | N | Baseline | Week 52 | Within group change | P value for change | N | | Baseline | Week 52 | Within group change | P value for change |  |
| BCVA | IVR | 19 | 59.5±8.8 | 78.3±9.7 | 18.8±8.2 | <0.0001 | 11 | | 58.9±7.7 | 75.2±13.1 | 16.3±10.3 | <0.0001 | 0.4681 |
|  | IVR+Laser | 18 | 55.1±11.4 | 73.8±10.9 | 18.7±11.0 | 0.0004 | 16 | | 53.7±7.9 | 71.2±9.5 | 17.5±8.8 | <0.0001 | 0.7250 |
| CRT（μm） | IVR | 19 | 515.2±144.8 | 229.6±43.2 | -285.6±144.6 | <0.0001 | 11 | | 517.6±193.7 | 245.6±89.0 | -272.0±240.1 | 0.0037 | 0.8474 |
|  | IVR+Laser | 18 | 638.9±235.1 | 243.4±48.6 | -395.6±245.0 | <0.0001 | 16 | | 495.8±189.0 | 258.6±114.4 | -237.3±212.3 | 0.0004 | 0.0540 |

^*^ Changes in BCVA and CRT from baseline to week 52 were normally distributed. T-test was adopted to compare changes in BCVA and CRT between patients with ischemia and those without ischemia in each treatment group.

**Appendix 8. Product-Limit Survival Estimates in group 1**

| **Time to 4thjection** | **Censored** | **Survival** | **Failure** | **Survival Standard Error** | **Number Failed** | **Number Left** |
| --- | --- | --- | --- | --- | --- | --- |
| 0.0000 |  | 1.0000 | 0 | 0 | 0 | 30 |
| 4.0000 |  | . | . | . | 1 | 29 |
| 4.0000 |  | 0.9333 | 0.0667 | 0.0455 | 2 | 28 |
| 8.0000 |  | . | . | . | 3 | 27 |
| 8.0000 |  | . | . | . | 4 | 26 |
| 8.0000 |  | . | . | . | 5 | 25 |
| 8.0000 |  | . | . | . | 6 | 24 |
| 8.0000 |  | . | . | . | 7 | 23 |
| 8.0000 |  | . | . | . | 8 | 22 |
| 8.0000 |  | 0.7000 | 0.3000 | 0.0837 | 9 | 21 |
| 12.0000 |  | . | . | . | 10 | 20 |
| 12.0000 |  | . | . | . | 11 | 19 |
| 12.0000 |  | . | . | . | 12 | 18 |
| 12.0000 |  | . | . | . | 13 | 17 |
| 12.0000 |  | 0.5333 | 0.4667 | 0.0911 | 14 | 16 |
| 24.0000 |  | 0.5000 | 0.5000 | 0.0913 | 15 | 15 |
| 32.0000 |  | 0.4667 | 0.5333 | 0.0911 | 16 | 14 |
| 40.0000 | ***** | . | . | . | 16 | 13 |
| 40.0000 | ***** | . | . | . | 16 | 12 |
| 40.0000 | ***** | . | . | . | 16 | 11 |
| 40.0000 | ***** | . | . | . | 16 | 10 |
| 40.0000 | ***** | . | . | . | 16 | 9 |
| 40.0000 | ***** | . | . | . | 16 | 8 |
| 40.0000 | ***** | . | . | . | 16 | 7 |
| 40.0000 | ***** | . | . | . | 16 | 6 |
| 40.0000 | ***** | . | . | . | 16 | 5 |
| 40.0000 | ***** | . | . | . | 16 | 4 |
| 40.0000 | ***** | . | . | . | 16 | 3 |
| 40.0000 | ***** | . | . | . | 16 | 2 |
| 40.0000 | ***** | . | . | . | 16 | 1 |
| 40.0000 | ***** | 0.4667 | . | . | 16 | 0 |

Note: The marked survival times are censored observations

**Appendix 9. Product-Limit Survival Estimates in group 2**

| **Time to 4thjection** | **Censored** |  | **Survival** | **Failure** | **Survival Standard Error** | **Number Failed** | **Number Left** |
| --- | --- | --- | --- | --- | --- | --- | --- |
| 0.0000 |  |  | 1.0000 | 0 | 0 | 0 | 34 |
| 4.0000 |  |  | . | . | . | 1 | 33 |
| 4.0000 |  |  | . | . | . | 2 | 32 |
| 4.0000 |  |  | . | . | . | 3 | 31 |
| 4.0000 |  |  | 0.8824 | 0.1176 | 0.0553 | 4 | 30 |
| 8.0000 |  |  | . | . | . | 5 | 29 |
| 8.0000 |  |  | . | . | . | 6 | 28 |
| 8.0000 |  |  | . | . | . | 7 | 27 |
| 8.0000 |  |  | . | . | . | 8 | 26 |
| 8.0000 |  |  | . | . | . | 9 | 25 |
| 8.0000 |  |  | . | . | . | 10 | 24 |
| 8.0000 |  |  | . | . | . | 11 | 23 |
| 8.0000 |  |  | . | . | . | 12 | 22 |
| 8.0000 |  |  | . | . | . | 13 | 21 |
| 8.0000 |  |  | . | . | . | 14 | 20 |
| 8.0000 |  |  | . | . | . | 15 | 19 |
| 8.0000 |  |  | 0.5294 | 0.4706 | 0.0856 | 16 | 18 |
| 12.0000 |  |  | . | . | . | 17 | 17 |
| 12.0000 |  |  | . | . | . | 18 | 16 |
| 12.0000 |  |  | . | . | . | 19 | 15 |
| 12.0000 |  |  | . | . | . | 20 | 14 |
| 12.0000 |  |  | . | . | . | 21 | 13 |
| 12.0000 |  |  | . | . | . | 22 | 12 |
| 12.0000 |  |  | 0.3235 | 0.6765 | 0.0802 | 23 | 11 |
| 16.0000 |  |  | 0.2941 | 0.7059 | 0.0781 | 24 | 10 |
| 40.0000 | ***** |  | . | . | . | 24 | 9 |
| 40.0000 | ***** |  | . | . | . | 24 | 8 |
| 40.0000 | ***** |  | . | . | . | 24 | 7 |
| 40.0000 | ***** |  | . | . | . | 24 | 6 |
| 40.0000 | ***** |  | . | . | . | 24 | 5 |
| 40.0000 | ***** |  | . | . | . | 24 | 4 |
| 40.0000 | ***** |  | . | . | . | 24 | 3 |
| 40.0000 | ***** |  | . | . | . | 24 | 2 |
| 40.0000 | ***** |  | . | . | . | 24 | 1 |
| 40.0000 | ***** |  | 0.2941 | . | . | 24 | 0 |

The marked survival times are censored observations

**Appendix 10. Time to fourth injection after the third injection between two groups.**


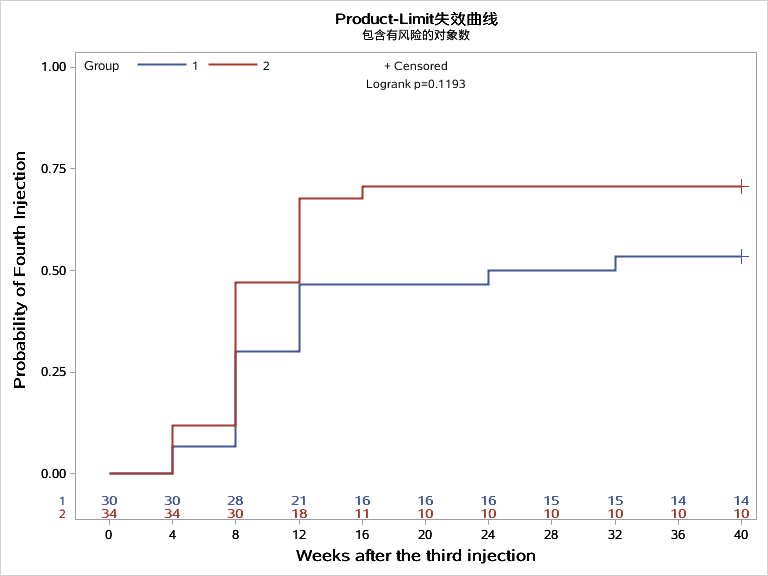


In Kaplan-Meier analysis, time to fourth injection did not differ significantly between groups (*P*=0.1193 in log-rank test). In Cox regression model with baseline BCVA adjusted, treatments in two groups was not statistically associated with fourth injection (Hazard Ratio 1.43, 95% CI: 0.75-2.76, *P*=0.2797). The proportional assumptions were met.
